# Perioperative Levosimendan Infusion in Patients With End-Stage Heart Failure Undergoing Left Ventricular Assist Device Implantation

**DOI:** 10.3389/fcvm.2022.888136

**Published:** 2022-04-28

**Authors:** Mahmoud Abdelshafy, Hagar Elsherbini, Ahmed Elkoumy, Andrew J. Simpkin, Hesham Elzomor, Kadir Caliskan, Osama Soliman

**Affiliations:** ^1^Discipline of Cardiology, Saolta Healthcare Group, Health Service Executive, Galway University Hospital, Galway, Ireland; ^2^CORRIB Core Lab, National University of Ireland Galway (NUIG), Galway, Ireland; ^3^Department of Cardiology, Al-Azhar University, Cairo, Egypt; ^4^Department of Cardiology, Erasmus MC University Medical Center, Rotterdam, Netherlands; ^5^Islamic Center of Cardiology and Cardiac Surgery, Al-Azhar University, Cairo, Egypt; ^6^School of Mathematical and Statistical Sciences, National University of Ireland Galway, Galway, Ireland; ^7^Insight Centre for Data Analytics, National University of Ireland Galway, Galway, Ireland; ^8^CÚRAM Centre for Medical Devices, Galway, Ireland

**Keywords:** levosimendan, LVAD, right-sided heart failure, inotropes, mechanical circularity support, heart failure

## Abstract

Left ventricular assist device (LVAD) therapy has been instrumental in saving lives of patients with end-stage heart failure (HF). Recent generation devices have short-to-mid-term survival rates close to heart transplantation. Unfortunately, up to 1 in 4 patients develop a life-threatening right-sided HF (RHF) early post LVAD implantation, with high morbidity and mortality rate, necessitating prolonged ICU stay, prolonged inotropic support, and implantation of a right-ventricular assist device. Pre-operative optimization of HF therapy could help in prevention, and/or mitigation of RHF. Levosimendan (LEVO) is a non-conventional inotropic agent that works by amplifying calcium sensitivity of troponin C in cardiac myocytes, without increasing the intra-cellular calcium or exacerbating ischemia. LEVO acts as an inodilator, which reduces the cardiac pre-, and after-load. LEVO administration is associated with hemodynamic improvements. Despite decades long of the use of LVAD and more than two decades of the use of LEVO for HF, the literature on LEVO use in LVAD is very limited. In this paper, we sought to conduct a systematic review to synthesize evidence related to the use of LEVO for the mitigation and/or prevention of RHF in patients undergoing LVAD implantation.

## Introduction

Left ventricular assist devices (LVADs) have been proven to be effective in reducing morbidity and mortality in patients with end-stage heart failure (HF) (1). Furthermore, second and third-generation LVADs provide a significantly improved quality of life and lower complications compared to early generation devices, almost approaching mid-term heart transplant results. Unfortunately, early perioperative mortality remains high, mainly due to over 20% of LVAD patients developing right-sided heart failure (RHF), which is strongly associated with increased mortality, morbidity, prolonged ICU, and hospital stay (2). Overall, LVAD does not support the heart completely, so the ability of the right ventricle to provide sufficient output to fill the left heart remains essential. Therefore, optimization of patients in the pre-operative status, besides optimal decongestion, probably by pre-conditioning of the sick heart could mitigate and/or prevent RHF.

Levosimendan (LEVO) is a non-conventional inotropic agent that acts as a calcium sensitizer. It works by amplifying calcium sensitivity of troponin C, without increasing the intra-cellular calcium or exacerbating ischemia. LEVO acts as an inodilator, which reduces the cardiac pre-, and after-load. LEVO acts also as a potassium channel opener, which has an active metabolite (OR1896) that peaks approximately 80–90 h after administration and is associated with hemodynamic improvements that are sustained for a week (3). The advantages of LEVO include beneficial symptomatic, hemodynamic, and neurohormonal effects, and improved peripheral organ perfusion and renal function. Importantly, there is no effect attenuation in patients using beta-blockers (4), which is currently one of the main HF treatment agents. In early studies, LEVO has been shown to decrease mortality (5), improve hemodynamics and reduce symptoms. In two recent systemic reviews, our group clearly showed the incremental value of LEVO infusions in the setting of end-stage HF (6), and cardiogenic shock patients needing VA-ECMO support (7).

In this study, we sought to conduct a systematic review to synthesize evidence related to the use of LEVO for the prevention and/or mitigation of RHF in patients undergoing LVAD implantation.

This systematic review was performed and reported according to the Preferred Reporting Items for Systematic Reviews and Meta-Analyses (PRISMA) guidelines (8). From inception to December 27, 2021, all relevant items were identified in collaboration with a Librarian at the Erasmus University Medical Centre. We searched Embase, Medline Ovid, Web of Science, Cochrane CENTRAL register of trials, and Google Scholar for articles published until the date of search. Adult (≥18 years) patients with LVAD receiving intravenous LEVO infusions were included. We included all clinical studies containing ≥10 patients and published in the last 30 years. Case reports, editorials, reviews, studies included orally administered LEVO, and articles that are not in English language were excluded. Two researchers (HaE and MA) independently reviewed abstracts and full texts in an unblinded standardized manner. Disagreements between the researchers about whether to include a study were discussed and resolved before final approval. Furthermore, references in selected articles were independently cross-checked by the two researchers for other relevant studies.

**FIGURE 1:**
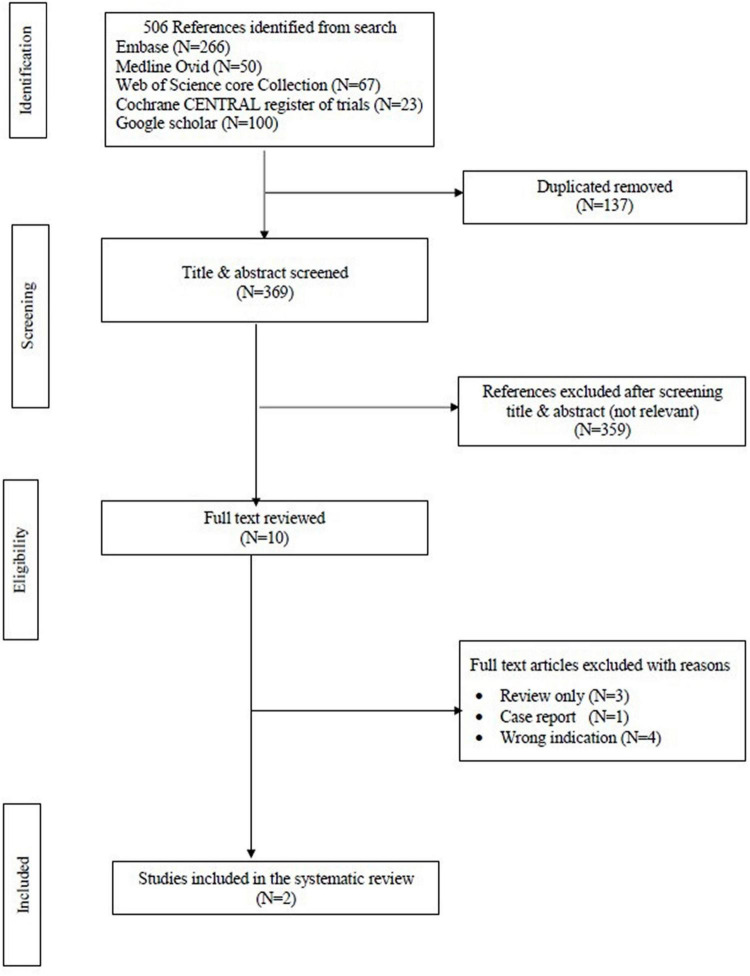
Preferred reporting items for systematic reviews and meta-analyses (PRISMA) flow chart of the data.

The search strategy resulted in 506 studies. After removal of duplicates, 369 studies remained. After reviewing the title and abstract, another 359 studies were removed due to irrelevance. Of the remaining 10 studies, only two met the pre-defined inclusion criteria and were consequently included in this review. Figure 1 displays the PRISMA flowchart. This systematic review included 106 patients from the two papers. A comparison between the two studies is shown in Table 1.

**TABLE 1:** Comparsion between the two studies included in the mini-review.

	Sponga et al. (1)	Kocabeyoglu et al. (9)
Journal, Year	ASAIO Journal, 2012	European Journal of Cardio-Thoracic Surgery, 2020
Type of study	Single-center study. NR.	Single-center study. Retrospective study.
Recruitment period	NR	May 2013 and October 2018
Inclusion criteria	LVAD patients with pre-operative borderline right ventricular function which was considered if one or more of the following echocardiographic criteria were unmet: 1. RV end-diastolic diameter <35 mm. 2. RV ejection fraction >30%. 3. Tricuspid regurgitation <grade II. 4. Short/long axis ratio of RV <0.6. 5. Pulmonary pressure <35 mm Hg.	Patients (age >18 years) with end-stage heart failure who underwent isolated LVAD implantation.
Exclusion criteria	Pre-operative use of centrifugal pump support.	1. Patients with INTERMACS class-1 profile. 2. Pre-operative ECMO support. 3. The need for a BIVAD. 4. LVAD implantation using the off-pump technique; and without pre-operative optimization.
Aim of the study	1. Examine the hemodynamic effect of levosimendan infusion in patients with borderline right ventricular function before urgent LVAD implantation. 2. Evaluate the prognostic effect of the response to levosimendan infusion.	Examine the hemodynamic effects of pre-operative levosimendan infusion in patients who underwent LVAD implantation and evaluate their prognoses.
1ry endpoint	NR	Early RHF.
2ry endpoint	NR	30-day and in-hospital mortality, need for RVAD, late RHF, CPB duration, ICU stay, and recovery of end-organ function.
Patients numbers/ characteristics	21 patients, Myocarditis (1 patient), DCM (7 patients), and ICM (13 patients).	85 patients, DCM (44 patients), and ICM (41 patients).
LVAD types	MicroMed DeBakey VAD, and Incor VAD.	HVAD (*n* = 51), HM II (*n* = 5), HM III (*n* = 28), Reliant Heart (*n* = 1).
RHF definition	Occurrence of two of the following criteria: Mean arterial pressure <55 mm Hg. Central venous pressure >16 mm Hg. Mixed venous saturation <55%. Cardiac index <2 L/min/m^2^. Inotropic support >20 units.	NR
Levosimendan protocol	0.1–0.2 ug/kg/min for a maximum of 48 h without bolus, 3 days before LVAD implantation.	0.1 ug/kg/min for a maximum of 48 h without a bolus, 3–10 days before LVAD implantation.
Patient cohorts	Group 1, patients who died due to RHF (*n* = 4, 19%). Group 2, included patients who survived or died from other reasons (*n* = 17, 81%).	Group A, levosimendan was administered in combination with other inotropes (*n* = 58, 86%). Group B, the same inotropes were administered without levosimendan (*n* = 27, 32%).
Results	The survival rate was 86% at 30 days and 57% at 1 and 2 years. Three patients underwent heart transplantation after a mean mechanical support time of 6 months. The main causes of death were RHF (4 patients), cerebral bleeding (3 patients), and sepsis (2 patients). Levosimendan improves pre-operative hemodynamic conditions in LVAD candidates with borderline RV function, and the response to levosimendan treatment helps to predict mortality and RHF.	The survival rates in groups A and B, respectively, were 77.2 and 73.1% at 30 days, 56.8 and 63.9% at 1 year and 46.4 and 53.2% at 3 years. 52 and 33 patients were bridged to transplant and destination therapy, respectively. The main causes of death were RHF (11 out of 20 patients, 55%), cerebrovascular accident (5 out of 20 patients, 25%; 3 patients with ischemic strokes, 2 patients with cerebral bleeding), and sepsis (4 out of 20 patients, 20%). The improvements in end-organ function were better in patients pre-conditioned with levosimendan; however, we found no difference between the 2 groups for the other outcomes.
RHF treatment	The four patients with RHF were treated with inhaled nitric acid, intravenous iloprost, and maximal inotropic support. No RVADs were implanted to treat RHF.	In group A, early RHF occurred in 15 out of 58 patients, 5 of these patients were treated with inhaled nitric oxide (with inhaled iloprost, if extubated), increased oral sildenafil (3 × 40 mg daily) and inotropic support—and RVAD implantation was needed in 10 patients unresponsive to medical treatment, 8 patients with ECMO and 2 patients with Levitronix (Abbott Inc., Chicago, IL, United States). In group B, early RHF was encountered in 5 patients (5 out of 27); only 2 patients responded to medical therapy and implantation of RVAD with ECMO was required in the remaining 3 patients.

*BIVAD, biventricular assist device; CPB, cardiopulmonary bypass; DCM, dilated cardiomyopathy; ECMO, extracorporeal membrane oxygenation; HM II, heart mate II, HM III, heart mate III; HVAD, heartWare ventricular assist device; ICM, ischemic cardiomyopathy; ICU, intensive care unit; INTERMACS, interagency registry for mechanically assisted circulatory support; LVAD, left ventricular assist device; NR, not reported; RHF, right sided heart failure; RV, right ventricle; RVAD, right ventricular assist device.*

Sponga et al. (1) reported the pre-operative use of LEVO in 21 LVAD patients at a single center. The LVADs used in this study were the MicroMed DeBakey VAD (MicroMed Technology, Inc., Houston, TX, United States) and the Incor VAD (Berlin Heart AG, Berlin, Germany).

The inclusion criteria was LVAD patients with pre-operative borderline right ventricular function which was considered if one or more of the following echocardiographic criteria were unmet: right ventricular end-diastolic diameter <35 mm, right ventricular ejection fraction >30%, tricuspid regurgitation <grade II, short/long axis ratio of right ventricle <0.6, pulmonary pressure <35 mm Hg. Pre-operative use of centrifugal pump support was considered an exclusion criteria.

Intravenous infusion of LEVO was administered in the intensive care unit at a dose of 0.1 to 0.2 ug/kg/min for a maximum of 48 h without bolus. The survival rate was 86% at 30 days and 57% at both 1 and 2 years following LVAD support. Four patients died because of RHF associated with low cardiac output and multiorgan failure, three patients died of cerebral bleeding and two patients died of sepsis. Patients were divided in two groups: group 1 (*N* = 4, 19%) included patients who died due to RHF and group 2 (*N* = 17, 81%) included patients who survived or died from other reasons. RHF was defined by the occurrence of two of the following criteria: mean arterial pressure <55 mm Hg, central venous pressure >16 mm Hg, mixed venous saturation <55%, cardiac index <2 L/min/m^2^, inotropic support >20 units.

**FIGURE 2:**
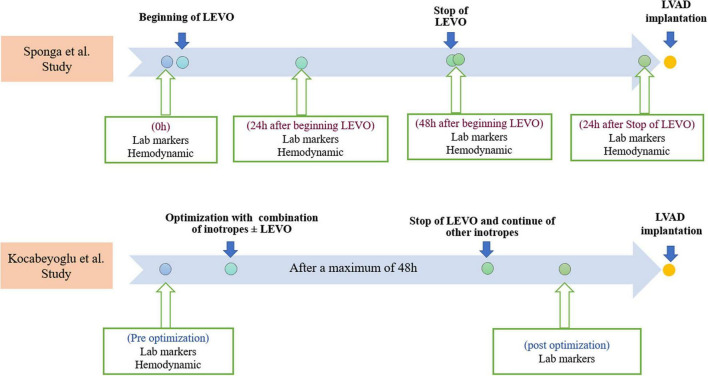
Timelines of Levosimendan infusions, laboratory markers, and hemodynamic measurements in both studies. Lab, laboratory; LEVO, levosimendan; LVAD, left ventricular assist device.

Furthermore, hemodynamic data, using a pulmonary artery catheter, and NT-proBNP values were collected four times which are summarized in Figure 2. After 48 h, LEVO infusion improved hemodynamic: The cardiac index increased in a significant and progressive manner by 21% (*p* = 0.014); pulmonary pressure decreased by 12% (*p* = 0.003); wedge pressure and central venous pressure both decreased by 15% (*p* = 0.028 and *p* = 0.016). There was no clear trend in pulmonary or systemic vascular resistances. Heart rate, systolic arterial pressure, mean arterial pressure, and diastolic arterial pressure did not change significantly during the 48 h treatment period. The changes in mixed venous oxygen saturation were significant only after 24 h (*p* = 0.008). However, the hemodynamic assessment during the time in the two groups was not statistically significant.

Regarding the NT-proBNP, the Median value at 72 h increased by 3% compared to time 0 in group 1 and decreased by 39% in group 2 (*p* = 0.008). In every single patient, a reduction of <25% at 72 h is a predictor of mortality with a sensitivity of 100% and specificity of 75%. NT-proBNP value after 48 h of treatment was significantly (*p* = 0.019) higher (8797 vs. 6733 pg/ml) in group 1 than in group 2.

However, 24 h after the end of the treatment hemodynamic performance was worse than baseline in the group who died because of RHF. In contrast, the hemodynamic improvement in patients who survived or died because of other reasons persisted longer. Therefore, the worsening of hemodynamic parameters despite the use of LEVO in RHF borderline patients is likely a marker of poor outcome in LVAD patients.

The second study by Kocabeyoglu et al. (9) was a retrospective single-center study that included 85 patients with end-stage HF who underwent isolated LVAD implantation. Patients with an Interagency Registry for Mechanically Assisted Circulatory Support (INTERMACS) class-1 profile; pre-operative extracorporeal membrane oxygenation (ECMO) support; the need for a biventricular assist device and LVAD implantation using the off-pump technique; and without pre-operative optimization were excluded. The LVADs used in this study are shown in Table 1. The patients were divided into two groups: the LEVO group (*N* = 58) included patients who received LEVO infusion at a rate of 0.1 ug/kg/min for a maximum of 48 h without a bolus, 3–10 days before LVAD implantation in addition to other inotropes. The no-LEVO group (*N* = 27) included patients who received conventional inotropic support without LEVO. LEVO was administered in combination with dobutamine (0–10 ug/kg/min), dopamine (0–8 ug/kg/min), milrinone (0–0.5 ug/kg/min) and norepinephrine (0–0.5 ug/kg/min) in the LEVO group. The same inotropes were administered in the no-LEVO group.

Post optimization (pre-LAVD implantation) with inotropic therapy, hepatic and renal functions and serum albumin values improved in both groups. However, the improvement was better in the LEVO group than in the no-LEVO group, although 30-day and in-hospital mortality was similar in both groups. Likewise, no significant differences were seen between both groups in terms of early RHF, need for right ventricular assist device (RVAD) or late RHF.

In both studies (1, 9), the administration of LEVO was safe and well-tolerated without signs of arrhythmia, tachycardia or hypotension. There were also no cardiac arrest events recorded, and the administration of LEVO was not interrupted because of side effects. Both studies show that LEVO can be successfully administered before LVAD implantation.

In Sponga et al., LEVO improved pre-operative hemodynamic conditions in LVAD candidates. Furthermore, the hemodynamic changes after LEVO infusion could help in predicting the mortality and RHF along with the baseline hemodynamic and echocardiographic data.

In addition, Kocabeyoglu et al. showed that perioperative optimizations of LVAD candidate improved hemodynamic conditions and thus improved end organ functions. Furthermore, the improvements in end organ function were better in patients preconditioned with LEVO, particularly renal function. This emphasizes earlier reports that LEVO preserves renal perfusion and glomerular filtration rate (9).

In a recently published meta-analysis by our group, LEVO use in patients undergoing ECMO was associated with significant VA-ECMO weaning success and lower risk of mortality (7). In addition, another meta-analysis by our group demonstrated that LEVO use in ambulatory patients with refractory HF has been associated with wide range of improved hemodynamics, echocardiographic parameters, reverse LV remodeling, lower filling pressures, and lower biomarkers of LV failure (6). On the other hand, long-term treatment with conventional intravenous inotropes increases mortality (6). More recently, Yalcin et al. (10) reported a successful use of intermittent LEVO infusion for treatment of a late RHF patient post LVAD.

These data on LEVO use in LVAD, although limited, are encouraging and suggest that there is at least hemodynamic improvements alongside improved organ perfusion associated with the use of LEVO in patients undergoing LVAD. The lack of survival benefits in these two studies could be due to very small number of patients involved in these studies. This in turn emphasizes the need for initiation of a large-scale randomized clinical trial to ascertain the clinical benefits of using LEVO in LVAD patients.

## Conclusion

In conclusion, current evidence of the use of Levosimendan in LVAD patients is very limited. So far, no survival benefits have been shown for the use of Levosimendan in LVAD patients, most probably due to underpowered studies. Therefore, further investigation, involving an adequately powered multicenter, randomized placebo-control study is warranted. In this proposed study, patients undergoing LVAD implantation and at risk for RHF should be randomized to receiving Levosimendan or placebo on top of the guideline-directed therapy. The primary safety endpoints should at least include the occurrence of arrhythmia, hypotension, tachycardia, termination of Levosimendan due to side effects. Efficacy endpoints should include at least, all-cause death, early and late RHF, hepatic dysfunction, renal dysfunction, duration of ICU stay, duration of hospital stay and hemodynamic improvements.

## Author Contributions

OS and KC: conceptualization, methodology, and supervision. MA and HaE: studies screening, data extraction. MA, HaE, KC, and OS: writing—original draft preparation. MA, KC, AE, HaE, HeE, AS and OS: writing—review and editing. All authors have read and agreed to the published version of the manuscript.

## Conflict of Interest

The authors declare that the research was conducted in the absence of any commercial or financial relationships that could be construed as a potential conflict of interest.

## Publisher’s Note

All claims expressed in this article are solely those of the authors and do not necessarily represent those of their affiliated organizations, or those of the publisher, the editors and the reviewers. Any product that may be evaluated in this article, or claim that may be made by its manufacturer, is not guaranteed or endorsed by the publisher.

## Abbreviations

CI: cardiac index
HF: heart failure
LEVO: levosimendan
LVAD: left ventricular assist implantation device
MCS: mechanical circulatory support
PP: pulmonary pressure
PRISMA: preferred reporting items for systematic reviews and meta-analysis
RHF: right-sided heart failure
RVAD: right ventricular assist device
RVF: right ventricular failure
VP: venous pressure
WP: wedge pressure.
